# Through the dark continent: African trypanosome development in the tsetse fly

**DOI:** 10.3389/fcimb.2013.00053

**Published:** 2013-09-18

**Authors:** Brice Rotureau, Jan Van Den Abbeele

**Affiliations:** ^1^Trypanosome Cell Biology Unit, Institut Pasteur and CNRS URA 2581Paris, France; ^2^Unit of Veterinary Protozoology, Department of Biomedical Sciences, Institute of Tropical MedicineAntwerp, Belgium; ^3^Laboratory of Zoophysiology, Department of Physiology, University of GhentGhent, Belgium

**Keywords:** African trypanosomes, development, parasite cycle, tsetse fly, vector

## Abstract

African trypanosomes are unicellular flagellated parasites causing trypanosomiases in Africa, a group of severe diseases also known as sleeping sickness in human and *nagana* in cattle. These parasites are almost exclusively transmitted by the bite of the tsetse fly. In this review, we describe and compare the three developmental programs of the main trypanosome species impacting human and animal health, with focus on the most recent observations. From here, some reflections are made on research issues concerning trypanosome developmental biology in the tsetse fly that are to be addressed in the future.

## Introduction

African trypanosomiases are a set of vector-borne diseases of humans and their livestock, which have devastating socio-economic consequences for the Sub-Saharan African continent. They result from infections with flagellated unicellular parasites named African trypanosomes (Kinetoplastida: Trypanosomatidae) of which the majority is exclusively transmitted by the bite of tsetse flies (Diptera: Glossinidae). Two species of African trypanosomes are responsible for Human African Trypanosomiasis (HAT), also known as sleeping sickness, whereas at least seven other species cause Animal African Trypanosomiasis (AAT) or *nagana* (Table 1).

**Table 1:** **Vectors and reservoirs of the African trypanosome species cyclically transmitted by tsetse flies**.

**Parasites**					**Main hosts**								
**Subgenus**	**Group**	**Species**	**Size (in microns)**	**Socio-economic impact**	**Vectors**				**Reservoirs**				
					**Transmission**	**Species**	**Development**	**Duration**	**Humans**	**Domestic animals**	**Wild animals**	**Experimental animals**	**Diseases**
*Dutonella*	*Vivax*	*T. vivax*	20–26	++	vectorial and mechanical (tabanids and mucids)	*G. morsitans spp., G. longipalpis, G. palpalis, G. tachinoides, G. pallidipes*	Proboscis	1 week	No	Cattle, sheep, goats, domestic buffalo, horses	Ruminants and equids	Goats, (mice)	AAT/nagana (souma/gobiat)
		*T. uniforme*	12–20	+		?				Cattle, sheep, goats	Ruminants	?	AAT/nagana
*Nannomonas*	*Congolense*	*T. congolense*	11–41	+++	vectorial	*G. morsitans spp., G. longipalpis, G. pallidipes, G. palpalis spp., G. tachinoides, G. brevipalpis*	Midgut and proboscis	2 weeks	No	Cattle, camels, horses, dogs, cats, sheep, goats, pigs, horses	Several groups	Rats, mice, guinea pigs, rabbits, goats	AAT/nagana (ghundi)
		*T. godfreyi*	9–22	?		?				Pigs	Suids	?	AAT/chronic nagana
		*T. simiae*	9–24	?		*G. morsitans spp., G. brevipalpis*				Pigs	Suids and primates	Rabbits, monkeys	AAT/acute nagana
*Picnomonas*		*T. suis*	13–19	?	vectorial	*G. morsitans spp., G. brevipalpis*	Midgut, salivary glands and proboscis	3 weeks	No	Pigs	Suids and primates	?	AAT/surra
*Trypanozoon*	*Brucei*	*T. brucei brucei*	11–39	++	vectorial	*G. morsitans spp., G. palpalis spp., G. pallidipes, G. tachinoides, G. brevipalpis*	Midgut, foregut and salivary glands	3 weeks	No	Horses, camels, dogs, sheep, goats, cattle, pigs, horses	Cattle, sheep, goat, pigs, horses, camels, dogs	Rats, thicket rats, mice, guinea pigs, rabbits	AAT/nagana (aina/baleri)
		*T. brucei rhodesiense*	12–42	+++		*G. morsitans spp., G. swynnertoni, G. pallidipes, G. fuscipes spp.*			Yes	?			HAT/acute sleeping sickness
		*T. brucei gambiense*	12–35	+++		*Palpalis group: G. palpalis spp., G. fuscipes spp., G. tachinoides*			Yes				HAT/chronic sleeping sickness

*Note that T. vivax was also previously known as T. cazalboui, T. caprae, T. angolense or T. bovis, and T. b. brucei as T. togolense, T. elephantis, T. pecaudi, T. anceps, T. ugandae or T. dukei*.

*?, Not determined*.

*Trypanosoma brucei rhodesiense* and *T. b. gambiense* are the causative agents of HAT in East/Southern Africa and West/Central Africa, respectively. The *T. b. rhodesiense* transmission cycle mainly involves wild and domestic animals, but intensified human-to-human transmission may occur during epidemics. The *T. b. gambiense* transmission cycle is mostly from human to human with occasional involvement of an animal reservoir. There are no prophylactic drugs or vaccines available for HAT and the few available treatments present a complex posology and severe side effects (Fevre et al., 2006; Brun et al., 2009). It is estimated that ~70 million people living in tsetse fly-infested areas are at different levels of risk of contracting HAT, especially in countries such as the Democratic Republic of Congo (DRC), Angola, South-Sudan, and the Central African Republic (Simarro et al., 2008, 2010, 2012; WHO, 2012). In 2011, less than 10,000 new cases were reported (Simarro et al., 2012), but probably more cases remained undetected given that sleeping sickness occurs in remote rural areas.

While these African trypanosome species are important for public health, other species cause severe disease in livestock (Table 1). *T. vivax* and *T. congolense* are the major pathogens of cattle and other ruminants, while *T. simiae, T. godfreyi*, and *T. suis* cause high mortality in domestic pigs. AAT restricts agricultural development on the African continent despite the availability of prophylactic and curative drugs. Moreover, it is worrying to see drug effectiveness being seriously threatened by an increasing drug resistance in animal trypanosomes (Delespaux et al., 2008).

In contrast to sand flies and mosquitoes, both male and female tsetse flies are obligatory blood feeders and are able to transmit trypanosomes. All pathogenic African trypanosomes are called Salivarian as they are transmitted via the saliva during the fly feeding. Tsetse flies are the exclusive cyclical insect vectors and it can be assumed that all species of *Glossina* could act as vectors (Table 1). Therefore, vector-oriented control is one of the main pillars in the fight against HAT and AAT to reduce parasite transmission and dissemination. In addition, direct mechanical transmission by other haematophagous flies such as tabanids and *Stomoxys* frequently occurs for *T. vivax*.

A comprehensive understanding of the trypanosome developmental pathway in the tsetse fly and the interactions that affect this journey is of high importance that will allow a better understanding of the transmission dynamics of these parasites in the natural context and the improvement of current transmission control measures. Recent reviews already present in-depth overviews of our knowledge on the tsetse-trypanosome interactions (Aksoy et al., 2003; Roditi and Lehane, 2008; Walshe et al., 2009), especially for *T. b. brucei* (Sharma et al., 2009; Dyer et al., 2013). Additionally, recent experimental work demonstrated the importance of microbiome-associated tsetse fly immunity in trypanosome development (Weiss et al., 2012, 2013). In this mini-review, we will focus on the major advancements during the past 5 years in our understanding of the trypanosome developmental programs inside the tsetse fly. We will compare the life cycles of the three epidemiologically most relevant trypanosome species, namely *T. vivax*, *T. congolense*, and *T. brucei.*

## African trypanosome developmental cycles in the tsetse fly

**Figure 1:**
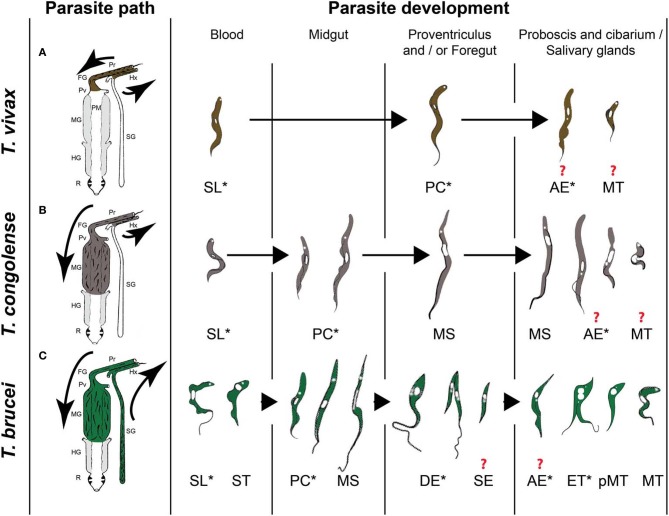
**The three types of African trypanosome development in the tsetse fly. (A)** *T. vivax* group. **(B)** *T. congolense* group. **(C)** *T. brucei* group. Parasite paths in the tsetse digestive tract are schematically presented in the left panel [adapted from (Hoare, 1972)]. Successive parasite stages found in the different organs are presented in a chronological order in the right panel [adapted from (Hoare, 1972; Peacock et al., 2007, 2012; Rotureau et al., 2012)]. ^*^ indicate proliferating stages and ? indicate an uncertainty with respect to the type of division and/or the transitional forms involved at this stage of development. Pr: proboscis, FG: foregut, Pv: proventriculus, PM: peritrophic matrix, MG: midgut, HG: hindgut, R: rectum, Hx: hypopharynx, SG: salivary glands, SL: slender trypomastigote, ST: stumpy trypomastigote, PC: procyclic trypomastigote, MS: mesocyclic trypomastigote, DE: long dividing epimastigote, SE: short epimastigote, AE: attached epimastigote, ET: epi-trypo dividing epimastigote, pMT: pre-metacyclic trypomastigote, MT: metacyclic trypomastigote.

A tsetse fly picks up trypanosomes during the acquisition of a blood meal on an infected mammal. Within the fly, the parasites have to go through a developmental cycle that can be simple or complex depending on the trypanosome species, consisting of several steps of proliferation, migration and differentiation. The final goal is to differentiate into infective metacyclic trypanosomes that are ready for transmission to the next mammalian host. The completion of this developmental cycle may take from a few days for *T. vivax* up to 3 weeks for *T. brucei*. It requires specific adaptations of the parasite to the different tsetse fly microenvironments, involving metabolic, cell surface protein or striking morphological modifications. Three distinct developmental programs have been described among Salivarian trypanosomes according to the complexity of their journey in the tsetse alimentary tract (Table 1 and Figure 1). Development of parasites of the *T. vivax* group (subgenus Duttonella) is restricted to the tsetse proboscis and cibarium. These parasites are believed to be the most ancient of the Salivarian trypanosomes. Trypanosomes of the Nannomonas subgenus comprise three species (including the economically important *T. congolense*) that successively develop in the midgut, foregut and proboscis of the flies. The *T. brucei* group (Trypanozoon subgenus) contains three trypanosome species (including the human-pathogenic *T. b. gambiense* and *T. b. rhodesiense)* that successively develop in the tsetse midgut, foregut, proboscis, and salivary glands. A remarkable common feature that occurs in three developmental programs is the switch between two morphotypes, i.e., the trypomastigote and epimastigote morphotype. These morphotypes are defined according to the relative position of the kinetoplast (condensed mitochondrial DNA) to the nucleus (Hoare and Wallace, 1966). In trypomastigotes, the kinetoplast localizes between the nucleus and the posterior end of the cell, whereas in epimastigote forms it is positioned at the other side, i.e., between the nucleus and the anterior end of the cell. This morphotype switch implies a drastic internal re-organization of the nucleus and kinetoplast that is assumed to be a costly cellular event. Therefore, the occurrence of this switch in all these cyclical developmental programs suggests that it plays an essential role in the parasite life cycle. The further very distinct developmental pathways of the three different trypanosome groups could be the result of a long-term co-evolution between parasites and their vectors that minimizes inter-trypanosome competition within the tsetse fly in order to maximize their respective transmission.

## Part I: The epimastigote's rise

In *T. vivax* and *T. congolense*, a single type of trypomastigote is found to proliferate in the bloodstream of the mammalian host. In contrast, two distinct types of *T. brucei* bloodstream parasites are distinguished: the dividing slender trypomastigote and the non-dividing tsetse-infective stumpy trypomastigote that is observed at peaks of parasitemia. Few hours after ingestion, *T. brucei* and *T. congolense* bloodstream trypomastigotes differentiate into procyclic trypomastigotes in the posterior midgut of the fly where they start multiplication (Figure 1). A number of these procyclics cross the peritrophic matrix, progressively elongate and migrate to the anterior part of the midgut as non-proliferative long mesocyclic trypomastigotes (Figure 1). Once in the proventriculus (cardia), *T. brucei* mesocyclic cells become thinner and adopt an epimastigote configuration accompanied by the migration of the nucleus to the posterior side of the kinetoplast (Sharma et al., 2008). This phenomenon was perturbed upon forced expression of the stress granule-associated protein ALBA3, showing for the first time the involvement of an RNA-binding protein in trypanosome development (Subota et al., 2011). Next, the long “tadpole-like” or “spermatozoa-like” epimastigotes start to divide asymmetrically in the proventriculus and foregut to produce a long and a short epimastigote (Van Den Abbeele et al., 1999) (Figure 1C). This dividing stage does not display any increase in cell volume neither in cell length, but is marked by a profound remodeling of the posterior part of the cytoskeleton and by changes in the molecular composition and/or organization of the flagellum attachment zone (Rotureau et al., 2011). In contrast to *T. brucei*, long *T. congolense* trypomastigotes (similar to the *T. brucei* mesocyclic forms) stop division in the proventriculus and become uniform in size. They retain a trypomastigote morphology during migration via the foregut to the proboscis where the trypomastigote-epimastigote transition takes place without any evident asymmetric division (Peacock et al., 2012) (Figure 1B). For *T. vivax*, only a small number of the ingested bloodstream forms remains in the foregut and cibarial region and undergoes a short developmental cycle from the trypomastigote into the epimastigote form (Moloo and Gray, 1989) (Figure 1A).

## Part II: The trypomastigote's revenge

For *T. congolense*, the long trypomastigotes in the foregut lumen migrate to the cibarium and proboscis and become epimastigotes that attach to the chitinous lining (rosette formation) where they proliferate and develop into infective metacyclics (Figure 1B). Dividing trypomastigotes and epimastigotes, as well as parasites in transition between the two morphotypes were observed at the same time in the proboscis (Peacock et al., 2012). Therefore, it remains uncertain whether these morphological transitions are mediated by cell division or by a differentiation event. *T. brucei* moves upstream from the proventriculus to the salivary glands via the foregut and proboscis while undergoing a first asymmetric division. Here, it is assumed that the long and highly motile epimastigote form acts as a transport vehicle that generates the short epimastigote in the salivary gland lumen by completing the asymmetric division (Figure 1C). Whether this process occurs continuously or only in a narrow time frame after parasites have reached the foregut is still under debate (Van Den Abbeele et al., 1999). However, based upon experimental work with tagged parasites, it is clear that only very few parasites (0–5) are successful in completing this migration process (Oberle et al., 2010).

Once in the salivary glands, the short epimastigote parasites attach to the epithelium via their flagellum and elongate (Tetley and Vickerman, 1985; Sharma et al., 2009) (Figure 1C). Recently, two distinct cycles of trypanosome proliferation were demonstrated to occur simultaneously in the salivary glands (Rotureau et al., 2012). The first cycle produces two equivalent cells that are attached to the epithelium. This mode of proliferation is predominant during the early phase of infection and ensures a rapid colonization of the glands. The second cycle is more frequent at a later stage and involves an asymmetric division (Figure 1C). It produces a daughter cell that matures into the infective metacyclic form that is released in the saliva, as demonstrated by the expression of specific molecular markers. It has been proposed that the coordination of these two alternative cell cycles contributes to the continuous production of infective parasites (Rotureau et al., 2012). In addition, *T. brucei* metacyclogenesis was shown to induce a severe modification of the tsetse salivary composition resulting in a drastically reduced anti-haemostatic potential and a hampered feeding performance, which could lead to an increased vector/host contact and parasite transmission in field conditions (Van Den Abbeele et al., 2010).

Although *T. vivax* development appears to be more simple, it remains poorly studied. Trypomastigote and epimastigote parasites from the foregut and cibarium migrate to the proboscis. Subsequent invasion of the hypopharynx by some of these forms leads to the further transformation into the infective metacyclic forms (Jefferies et al., 1987; Moloo and Gray, 1989). As for *T. congolense*, the exact developmental program from the trypomastigote to the epimastigote, and back to the trypomastigote morphotype remains to be unravelled: does it involve an asymmetric division as described for *T. brucei* or does it depend on a differentiation event?

It is clear that both *T. brucei* and *T. congolense* parasites go through a complex and tortuous developmental program in the tsetse fly vector. This strategy confers important advantages to the parasite such as multiple transmission opportunities to new mammalian hosts during the entire life of the tsetse fly as well as the opportunity for genetic (sexual) exchange (Aksoy et al., 2003).

## Sex and the EPI

*T. brucei* experiences a pronounced bottleneck during differentiation and migration from the midgut to the salivary glands (Oberle et al., 2010). An important outcome of this bottleneck is that rare variants can be amplified in individual flies and subsequently disseminated into the mammalian host population. This is compatible with the epidemic population structure of *T. brucei* in which clonal expansion of a few genotypes in a region occurs against a background of recombination between strains (Oberle et al., 2010). Indeed, beside the natural selection of variants, genetic exchange in *T. brucei* as well as *T. congolense* is occurring in the tsetse fly. The cellular mechanisms underlying this sexual stage in the tsetse fly remain to be elucidated. Recently, Gibson's lab demonstrated the existence of intraclonal mating during *T. brucei* fly transmission, in addition to the previously described interclonal recombination, which makes it unlikely that *T. brucei* remains genetically unaltered during fly transmission (Peacock et al., 2009). Homologs of meiotic genes were identified in the *T. brucei* genome and the expression of three functionally distinct meiosis-specific proteins in the nucleus of a specific epimastigote cell type has been demonstrated, defining a previously unidentified developmental trypanosome stage in the tsetse fly salivary glands (Peacock et al., 2011). Expression occurred in clonal and mixed infections, indicating that the meiotic program is an intrinsic but hitherto cryptic part of the developmental cycle of trypanosomes. In experimental crosses, expression of the meiosis-specific proteins was observed to occur before cell fusion (Peacock et al., 2011). However, the actual trypanosome fusion event has not yet been described.

It remains puzzling that the *T. brucei* parasite developed a complex sexual exchange mechanism in the tsetse salivary glands, knowing that in natural situations the probability of two different *T. brucei* strains successfully developing and meeting in the salivary glands is extremely low. Of course, the fact that even intraclonal mating results in recombination events that introduce genetic variability in the metacyclic trypanosomes could be considered as an evolutionary advantage. The probability of mating events for *T. congolense* in the tsetse fly are likely to be higher, as suggested by recent population genetics analyses where the observed parasite genotypic diversity could only be explained by the occurrence of frequent mating (Morrison et al., 2009). However, no experimental information on where this mating event occurs during development in the tsetse fly is yet available. For *T. vivax*, nothing is known so far about the existence of mating but the genetic diversity between populations is estimated to be low (Tait et al., 2011). The latter could result from the occurrence of non-cyclical transmission and/or the absence of genetic exchange during cyclical development in tsetse flies.

## Tsetse in a test-tube?

Unravelling the intricate interactions between African trypanosomes and the tsetse vector remains a challenge due to technical constraints and time consuming experimental procedures. Recently, by overexpressing a single RNA-binding protein, TbRBP6, in cultured non-infectious *T. brucei* trypanosomes, Kolev et al. (2012) *in vitro* reproduced the developmental stages that have been observed in tsetse, including the generation of infective metacyclic forms that express the variant surface glycoprotein. It can be stated that this recent finding is one of the major breakthroughs of the last decades in *T. brucei* research, opening avenues to experimentally unravel the parasite biology during the final development into the infective metacyclic stage. The developmental cycle of *T. congolense* can be reliably reproduced *in vitro* and cultures yield large numbers of trypanosomes of different life cycle stages (Coustou et al., 2010). Another advancement was also recently achieved by the optimization and standardization of non-infective *T. vivax* epimastigote axenic cultures leading to *in vitro* differentiation into metacyclic infective forms (D'Archivio et al., 2011). However, although these methods could provide an easy access to the intricate molecular and cellular events leading trypanosomes to the acquisition of infectivity, they will not replace the use of the tsetse fly infection model to unravel the complex cross-talks between these two organisms.

## Some reflections for future research

For *T. brucei*, our understanding of the developmental cycle in the fly has been steadily improving by information emerging from recent molecular and cell biological analyses (Sharma et al., 2009; Dyer et al., 2013). *T. congolense* shares a common migratory pathway with *T. brucei* but the transitional developmental stages in the foregut and mouthparts are remarkably different in these two trypanosome species (Peacock et al., 2012). Our knowledge on *T. vivax* development in the tsetse vector is currently very limited, but the increasing socio-economic impact of this widespread parasite prompts in-depth re-investigation of its life cycle.

A strikingly common feature of the three developmental programs is the passage through the epimastigote morphotype. Here, details of transitional forms are especially sparse for *T. vivax* and *T. congolense*. One of the key questions is whether there is any form equivalent to the asymmetric dividing stage of *T. brucei*. Moreover, the biological reason for this obligatory morphotype switch remains elusive. Is the epimastigote configuration more adapted to cell fixation via the flagellum compared to the trypomastigote? This flagellum attachment to a solid substrate is a prerequisite for multiple parasite transmission as it provides an efficient way to maintain a pool of progenitor cells that continuously produces infective forms without being expelled with the saliva during tsetse fly feeding.

Is the complex and directional development of *T. brucei* (and *T. congolense*) in the tsetse driven by an active parasite sensing? Indeed, this journey is highly organized in time and space where scanning of the different micro-environments by the parasite can be assumed to be essential for proper cell orientation during migration as well as for initiation of cell cycle switches and differentiation. During this travel through the dark continent, the trypanosome flagellum could act as a sensory organelle, especially through the MAP kinase pathway (Rotureau et al., 2009). Supporting this hypothesis, MAP kinase kinase 1 null mutants of a fly-transmissible strain were able to establish midgut infections at normal rates and intensities, but were incapable of colonizing the salivary glands, suggesting that the signaling cascades involving MAPKK1 are indispensable for transmission of *T. brucei* (Morand et al., 2012). The role of the *T. brucei* transmembrane protein PSSA-2 might also be to sense and transmit signals contributing to the parasite's decision to divide, differentiate or migrate (Fragoso et al., 2009). Whether transmembrane signaling molecules such as adenylate cyclases are actively involved in sensing and steering the parasite developmental program in tsetse is still an open question (Salmon et al., 2012). Recently, an intriguing phenomenon of social motility in African trypanosomes has been documented in *in vitro* experimental settings (Oberholzer et al., 2010) but the *in vivo* biological relevance inside the tsetse fly remains to be elucidated.

The recent publications of tsetse and trypanosome genomes as well as the development and refinement of molecular and cellular tools have paved the way for new functional approaches to study the African trypanosomes' development in their vectors. Morphological remodeling, motility, metabolism, control of differentiation and especially sensing are some of the most promising areas to identify targets to block trypanosome development and/or transmission.

### Conflict of interest statement

The authors declare that the research was conducted in the absence of any commercial or financial relationships that could be construed as a potential conflict of interest.
